# Reliability of Nationwide Prevalence Estimates of Dementia: A Critical Appraisal Based on Brazilian Surveys

**DOI:** 10.1371/journal.pone.0131979

**Published:** 2015-07-01

**Authors:** Flávio Chaimowicz, Alex Burdorf

**Affiliations:** 1 Department of Internal Medicine, Faculty of Medicine, Federal University of Minas Gerais, Belo Horizonte, Minas Gerais, Brazil; 2 Department of Public Health, Erasmus Medical Center, Erasmus University, Rotterdam, Netherlands; Cardiff University, UNITED KINGDOM

## Abstract

**Background:**

The nationwide dementia prevalence is usually calculated by applying the results of local surveys to countries’ populations. To evaluate the reliability of such estimations in developing countries, we chose Brazil as an example. We carried out a systematic review of dementia surveys, ascertained their risk of bias, and present the best estimate of occurrence of dementia in Brazil.

**Methods and Findings:**

We carried out an electronic search of PubMed, Latin-American databases, and a Brazilian thesis database for surveys focusing on dementia prevalence in Brazil. The systematic review was registered at PROSPERO (CRD42014008815). Among the 35 studies found, 15 analyzed population-based random samples. However, most of them utilized inadequate criteria for diagnostics. Six studies without these limitations were further analyzed to assess the risk of selection, attrition, outcome and population bias as well as several statistical issues. All the studies presented moderate or high risk of bias in at least two domains due to the following features: high non-response, inaccurate cut-offs, and doubtful accuracy of the examiners. Two studies had limited external validity due to high rates of illiteracy or low income. The three studies with adequate generalizability and the lowest risk of bias presented a prevalence of dementia between 7.1% and 8.3% among subjects aged 65 years and older. However, after adjustment for accuracy of screening, the best available evidence points towards a figure between 15.2% and 16.3%.

**Conclusions:**

The risk of bias may strongly limit the generalizability of dementia prevalence estimates in developing countries. Extrapolations that have already been made for Brazil and Latin America were based on a prevalence that should have been adjusted for screening accuracy or not used at all due to severe bias. Similar evaluations regarding other developing countries are needed in order to verify the scope of these limitations.

## Introduction

It is estimated that dementia affects 36 million people worldwide, 58% of whom are living in developing countries [1]. Cognitive and behavioral problems and progressive dependency considerably reduce the quality of life of these patients, disrupt family structures and strain societal resources [2]. Global societal costs of dementia care have reached US$ 604 billion in 2010 [3] entailing the need of accurate estimates for public health planning.

Nationwide figures of dementia are usually calculated by multiplying demographic (population) data with surveys’ prevalence [4]. Yet the accuracy of these estimates is intrinsically related to the reliability of the input data. For this reason, score systems have been developed to assess the methodological quality of these surveys [1, 5]. However, as the scores arbitrarily weight different component items (e.g., sampling techniques, response rates) their results are largely inconsistent [6]. Actually, these criteria should be used in instruments that systematically assess the *risk of bias* related to internal and external validity. Each study should present sufficient detail to support a judgment about the extent to which potential sources of bias have been avoided in different domains [7, 8]. To our knowledge, this method has not yet been applied to evaluate dementia surveys.

In Latin America, most of the high-quality epidemiological studies on dementia were conducted in Brazil, presenting direct evidence on the occurrence in different populations [1]. Many of these studies comply with internationally accepted criteria for sampling strategies, case ascertainment procedures and outcome definitions and have generated reliable data regarding specific groups and populations. These studies bring, therefore, the opportunity to analyze how and to what extent bias could impair generalizations to a nationwide estimate of dementia.

While some surveys with adequate sampling strategies utilized biased ascertainment procedures, others carried out highly accurate screening and diagnostics methods in non-random samples in the general population. Internal validity, for example, is a concern regarding a study which evaluated the prevalence of cognitive and functional impairment (CFI) among 870 community-dwelling older subjects in Brazil [9], since the 19.2% prevalence of CFI may be biased as 2/3 of the sample was composed by users of a neurology outpatient unit, which does not represent the general population. A related problem arose in another population-based survey which estimated a 18.9% prevalence of CFI [10]. Although this time a random sample was evaluated, 37% of the 1,828 eligible subjects refused to participate. Even when the representativeness is assured, the results may be biased when the diagnosis of dementia relies on non-validated tests, as was the case in the 13.8% prevalence among 875 older subjects from a population-based random sample who were classified according to their scores on the *Brazil Old Age Schedule questionnaire* [11]. Even when internal validity procedures meet standard requirements, limited external validity may compromise the generalizability of the results. An illustration of this problem is a survey which estimated a 16.9% prevalence of dementia among 683 older subjects from a health insurance plan in Rio de Janeiro [12].

In the present study we evaluated how and to what extent bias may hinder the utilization of results from dementia surveys in developing countries to arrive at a reliable estimate of the nationwide prevalence of dementia. With this aim we carried out a systematic review of dementia surveys in Brazil, ascertained the risk of bias in these surveys, and presented a final estimate of dementia in Brazil.

## Methodology

This investigation was performed in three steps: first we carried out a systematic review to identify Brazilian population-based surveys with acceptable sampling methods which presented dementia prevalence estimates. Second, we developed a multi-domain checklist to evaluate the risk of bias of the selected studies. Third, we ascertained to what extent the selected surveys were suitable for nationwide generalizations and presented a final estimate of dementia in Brazil.

### Systematic Review

Methods of the systematic review were specified in advance, documented in a protocol (S1 Protocol) following PRISMA guidelines [8] (S1 PRISMA Checklist) and registered at PROSPERO—International prospective register of systematic reviews (CRD42014008815). We sought and included relevant literature in English and Portuguese regarding population-based studies of dementia prevalence in Brazil. With the terms “dementia”, “Alzheimer”, “prevalence”, “epidemiology”, “Brazil” and the correspondent terms in Portuguese we carried out an electronic search of PubMed (since 1984), two Latin-American databases (LILACS and SciELO, since inception) and the Brazilian thesis databases; all reference lists were also scanned (S1 File).

**Fig 1:**
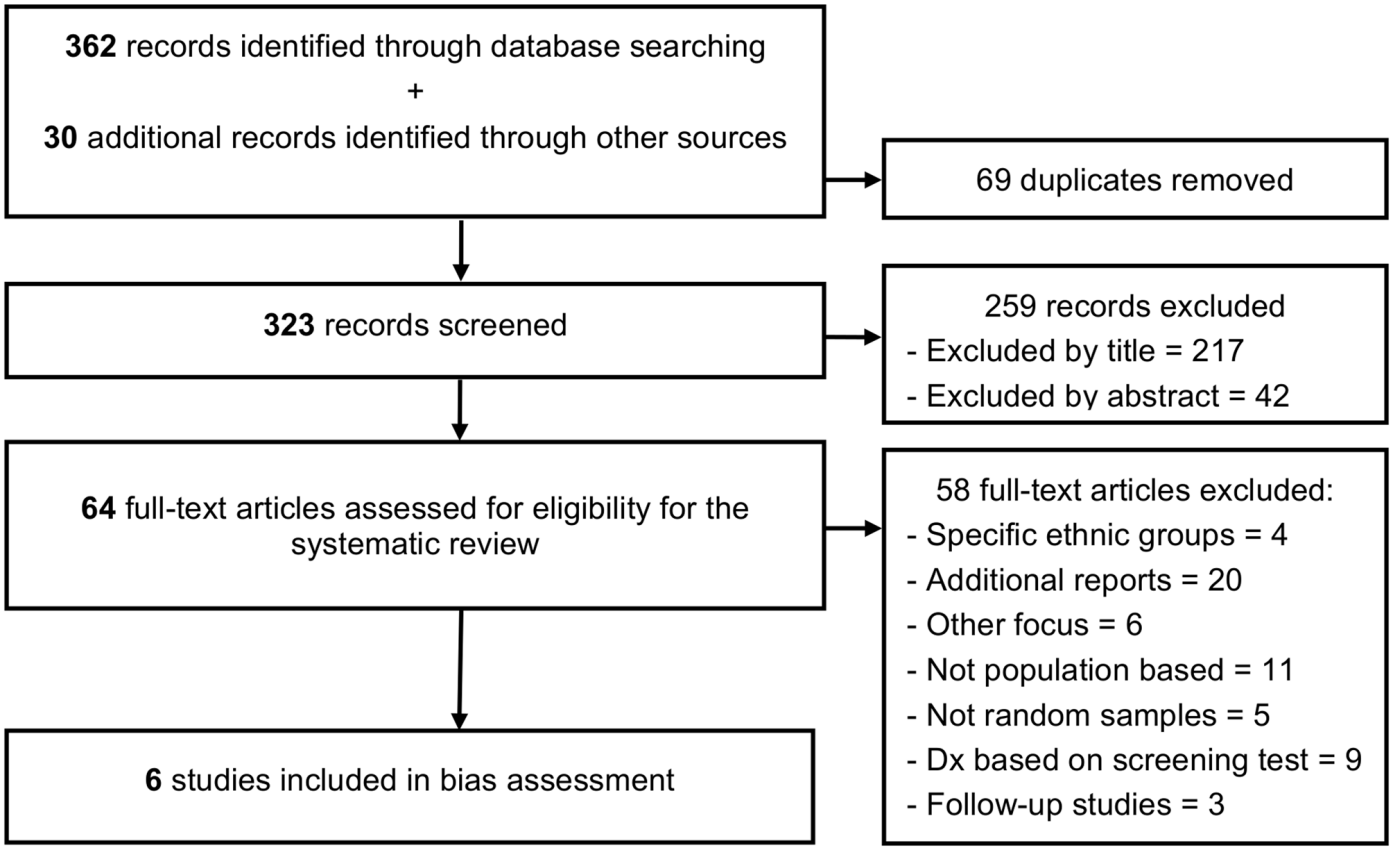
Flow-chart of the systematic review.

The search resulted in 323 records after 69 duplicates of the same reports were excluded (Fig 1). Title and abstracts were screened and 259 articles not related to “dementia”, “prevalence” and “Brazil” were removed, notwithstanding attempts to be over-inclusive at this stage. Next, an eligibility assessment of the remaining full-texts was performed in a standardized manner by one of the authors (FC). At this step we excluded articles assessing specific ethnic groups or focusing on specific types of dementia (e.g., vascular dementia), those not addressing entire populations or random samples (which mainly evaluated hospital and ambulatory service users), studies with non-specific diagnostic criteria (e.g., “cognitive impairment”) or diagnosis solely based on screening tests, and finally studies of cohort follow-ups or additional reports of already selected surveys (S2 File).

### The multi-domain checklist to evaluate the risk of bias

We used a checklist for critically evaluating the risk of bias in the six studies potentially eligible for a meta-analysis and subsequent nationwide estimate (S3 File Its content was entirely based on the criteria proposed by Loney et al. [4] and used by Prince et al. [1]. It is also in agreement with current recommendations for observational studies and systematic reviews [6, 7, 8, 13]. We considered that, specifically focusing on dementia surveys, the checklist should address four internal validity domains (selection, attrition and outcome bias as well as some statistical analysis issues) and also population bias, which mainly compromise external validity. These sources of bias are described below.

To avoid **selection bias**—systematic differences between characteristics of the sample and the source population—the best sampling method is random sampling from census data of the whole population. Stratification by age is also advisable in dementia surveys since the small group of octogenarians is the one that presents the higher prevalence. Adequate inclusion and exclusion criteria as well as cluster sampling by income groups may additionally enhance the representativeness of the sample.

**Attrition bias** may arise due to systematic differences between groups in withdrawals: the non-response may be associated to dementia as well as to healthy states (e.g., the healthy subjects went to work and were absent from home). It is considered acceptable if non-response is lower than 30% [5] or 40% [1]. The risk of bias may be minimized if the reasons for non-response are identified and are not associated with dementia, or if the characteristics of the non-responders are comparable to those included in the study. For this last reason, it may be advantageous to obtain baseline measurements for all participants recruited, since it enables the analysis of immediate drop-outs, which are relatively common among older age groups due to hospitalization or death.

**Outcome bias** is especially relevant in the field of dementia surveys since there are no biological markers to establish the diagnosis. Internationally accepted criteria (e.g., the Diagnostic and Statistical Manual of Mental Disorders, 4th Edition [DSM-IV]) should be used and these generally require that the declining memory is accompanied by deficits in other intellectual functions leading to social or occupational impairment. Therefore, the diagnosis shall not rely solely on cognitive tests but rather include a comprehensive neuropsychiatric test as well as a disability assessment and informant interview, and be complemented by clinical evaluation to exclude other causes (e.g., depression). Consequently, additional sources of bias are the utilization of non-validated methods for screening and clinical evaluation and the inadequate training and reliability of assessors.

To save time and costs, many studies begin with a screening phase and reserve the multi-domain assessment for the screen-positive subjects. This procedure not only increases the risk of attrition bias, but also brings about a relevant statistical issue, i.e. the need to back-weight the prevalence found in the 2^nd^ phase with the accuracy of the screening. This adjustment may be done by the formula (%S+)*(PPV) + (%S-)*(1-NPV) which weighs the proportion of screen-positive (%S+) and negative subjects (%S-) with the positive and negative predictive values (PPV, NPV). For this reason, a sample of the screen-negative subjects must also undergo the 2^nd^ phase evaluation in order to enable the calculation of the NPV.

**Population bias** may occur if there are differences between the source population of the original sample and the population of the whole country. It is a major concern regarding nationwide estimations of dementia prevalence and is mainly related to characteristics such as age, sex, ethnicity, and literacy levels.

The checklist developed to evaluate the risk of bias was pilot tested with one study, modified accordingly and applied to the remaining five surveys. The risk of bias in each domain was then classified as low, moderate (enabling adjustments/interpretation) or high (preventing the nationwide estimations).

## Results

### Bias assessment

The main characteristics of the six population-based surveys which met the inclusion and exclusion criteria are presented in Table 1. All but one evaluated large samples (> 1,000) and half of the studies included only subjects aged 65 years and older. Although the proportion of octogenarians was quite similar, the illiteracy rates varied from 10.1% to 50.9%. Four studies were two-phase studies and only one study did not apply the DSM-IV criteria for the outcome.

**Table 1:** Characteristics of the studies selected for the qualitative analysis.

Survey	N	Age	>80 years (%)	Illiteracy (%)	Sampling	Methods	Dementia criteria
Herrera et al. 2002 [22].	1,656	65+	18.5	34.2	Census based systematic sample	1^st^ phase: screening (MMSE; PFAQ); 2^nd^ phase: clinical and neurological evaluation, lab tests, brain scan.	DSM-IV
Ramos-Cerqueira et al. 2005 [15].	2,222	65+	17.6	Not stated	All individuals routinely visited by health care workers	1^st^ phase: case finding protocol; 2^nd^ phase: evaluation by a psychiatrist	DSM-IV
Magalhães et al. 2008 [14].	466	60+	18.9	50.9	All individuals living in the village	Standardized questionnaire, neurological evaluation, CAMDEX	CAMDEX
Scazufca et al. 2008 [17].	2,072	65+	13.6	38.3	All individuals living in a low income area	Cognitive, functional and limited neurological evaluation, interview with informant	DSM-IV
Bottino et al. 2008 [19].	1,563	60+	16.5	15.6	Cluster census based random sample (high, medium, low income)	1^st^ phase: MMSE, FOME, IQCODE, B-ADL; 2^nd^ phase: clinical/neurological evaluation, CAMDEX, lab tests, brain scan	DSM-IV
Lopes et al. 2007 [10]/ 2012 [21].	1,145	60+	14.2	10.1	Cluster census based random sample (high, medium, low income)	1^st^ phase: MMSE, FOME, IQCODE, B-ADL; 2^nd^ phase: clinical/neurological evaluation, CAMDEX, lab tests, brain scan	DSM-IV

MMSE: Mini-Mental State Examination; PFAQ: Pfeffer Functional Activities Questionnaire; DSM-IV: Diagnostic and Statistical Manual of Mental Disorders, 4th edition; CAMDEX: Cambridge Examination for Mental Disorders; FOME: Fuld Object Memory Evaluation, IQCODE Informant Questionnaire on Cognitive Decline in the Elderly, B-ADL: Bayer-Activities of Daily Living Scale.

The results of the risk of bias assessment are presented in Table 2; all studies presented moderate or high risk of bias on at least two domains. The characteristics of the studies related to the risk of bias are described below.

**Table 2:** Risk of bias from the six articles selected for the qualitative analysis.

	Bottino et al. [19]	Lopes et al. [10, 21]	Herrera et al. [22]	Scazufca et al. [17]	Ramos-Cerqueira et al. [15]	Magalhães et al. [14]
**Internal validity**						
Selection bias	0	0	0	0	1	0
Attrition bias	1	1	0	0	1	0
Outcome bias	0	0	1	1	2	2
Statistical analysis issues	0	0	1	0	0	0
**External validity**						
Population bias	1	1	0	2	1	2

Risk of bias: 0 = low; 1 = moderate; 2 = high.

Magalhães et al. [14] surveyed a rural population, which does not represent the 84% of Brazilians who live in urban areas. There is no information regarding the training and accuracy of the geriatricians, cardiologists and neurologists who evaluated the subjects. Their estimates (49.7% prevalence of dementia) were probably inflated by an improperly high sensitivity of the Cambridge Cognitive Examination (CAMDEX). This instrument was designed to be used on individuals with positive results in screening tests, and not as screening test itself in one phase surveys. A further increase in the prevalence may have been caused by the large proportions of illiteracy (50.9%) and visual impairment (28.1%) in the sample, both of which are known factors associated to lower scores. Additionally, no laboratory tests or brain scans were done to exclude other causes of cognitive deficits.

Ramos-Cerqueira et al. [15] trained community health care workers to identify probable cases of dementia among the families they visited regularly. The training consisted of a three hour session with audiovisual resources followed by discussion. A psychiatrist evaluated 85% of the suspected cases (3.7% of 2,222 eligible) using DSM-IV criteria to confirm the clinical diagnosis of dementia. It is not clearly stated if the whole population of the town was included neither if the respondents were comparable to the 15% non-respondents (raising concerns regarding selection and attrition bias). There is also no description of the accuracy of the case finding procedures, which was previously developed and evaluated in India [16], but not validated in Brazil. The methods utilized by the psychiatrist to establish the diagnosis of dementia were also not presented.

Scazufca et al. [17] evaluated subjects living in areas with very low Human Development Indexes which narrows the generalizability of the results. Dementia was diagnosed by mental health workers through the 10/66 Dementia Research Group procedure. It comprises a structured neurological assessment and informant interview (regarding cognitive and functional decline), all of which lasting 90 minutes, but no laboratory work up or brain scans. The method was validated in Brazil using as gold standard local clinicians’ diagnosis according to DSM-IV and Clinical Dementia Rating mild-to-moderate dementia. It has shown a lower specificity among depressed older subjects in Latin America, as their relatives tend to rate them as cognitively or functionally impaired [18].

Bottino et al. [19] evaluated 70.0% of the 2,233 eligible subjects, the main reason for non-response being “*refusal*” (86%). The screening procedure had previously demonstrated high accuracy in Brazil when applied by a trained neurologist and psychiatrists in a reference ambulatory setting [20]. In the survey it was applied at the subjects’ homes by previously trained lay interviewers. The Mini-mental state examination (MMSE) literacy-specific scores used in the study were also different from those previously tested. From the 250 (16.0%) subjects positive at the screening phase, 164 (65.6%) were evaluated by “*a neurologist or psychiatrist of the group”* in the 2^nd^ phase, which included a translated version of CAMDEX. The main reason for non-response at the 2^nd^ phase was “*not found*” (67.0%). The screen-positive subjects, who were evaluated, however, were similar to those not evaluated regarding demographic data and results at the screening tests. The evaluation of a random sample with 20% of the screen-negative subjects identified 3.8% as dementia cases, enabling the calculation of the screening sensitivity (0.94), specificity (0.71), PPV (0.62) and NPV (0.96), for which the prevalence was adjusted.

Lopes et al. [10, 21], with exactly the same methodology as Bottino et al. [19], evaluated 62.7% of the 1,828 eligible subjects from Ribeirão Preto Municipality (São Paulo state) with the only cause for non-response being “*refusal*” (100%). From the 217 (18.9%) screen-positive subjects, 130 (60.6%) were examined by *“trained psychiatrists or geriatricians”*. The main reason for non-response was “*refusal*” (62.1%). The screen-positive subjects evaluated presented higher scores on MMSE and Fuld Object Memory Evaluation than those who were not evaluated. The evaluation of a random sample with 7.8% of the screen-negative subjects identified 3.3% as dementia cases, enabling the calculation of the screening sensitivity (0.99), specificity (0.32), PPV (0.52) and NPV (0.97), for which the prevalence was adjusted.

Herrera et al. [22] evaluated 98.7% of the 1,681 eligible subjects at Catanduva (São Paulo state). From the 234 (14.1%) screen-positive subjects identified by graduated medical students, 220 (94%) were assessed by a trained neurologist who subsequently discussed each case with two other trained neurologists. No evaluation of screen-negative subjects was done, but it was possible to estimate the PPV (0.54): 118 cases confirmed among 220 screen-positive subjects evaluated. The cut-off level for the screening with MMSE was lower than that used by Bottino and Lopes and the recommended level for the Brazilian population [23] which may have reduced its sensitivity. For this reason and for purposes of comparison, to calculate the adjusted prevalence in this paper we arbitrarily assumed a NPV of 0.90, which is 10% lower than that reported by the authors.

### Dementia prevalence

**Fig 2:**
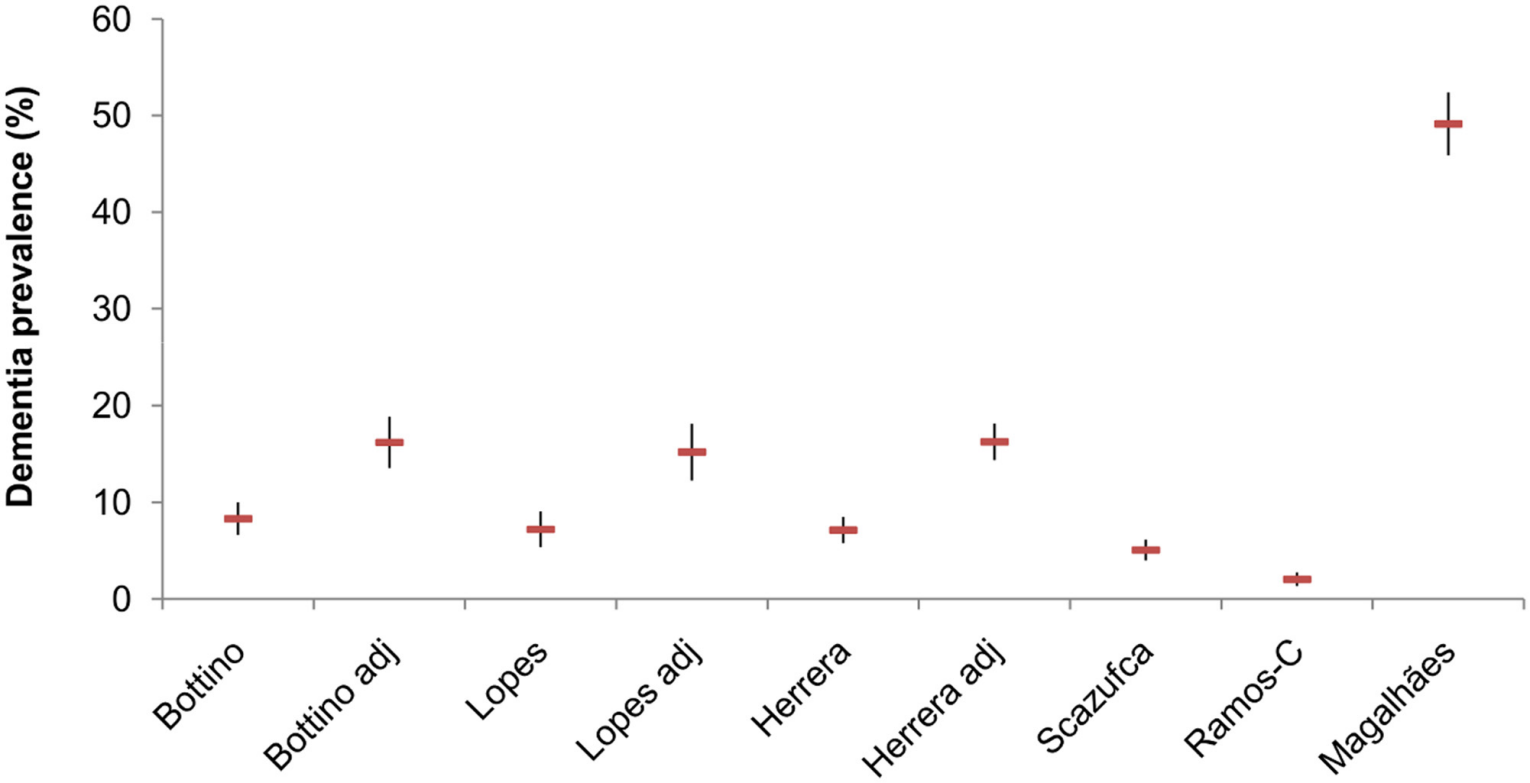
Overall crude and adjusted prevalences of dementia and 95% CIs among subjects aged 65 years* and older. * Magalhães: subjects aged 60 years and older.

The crude dementia prevalence among subjects aged 65 years and older found by the four studies with the lowest risk of bias were quite similar, varying from 5.1% to 8.3% (Fig 2; S1 Table). Adjusting the prevalence for the screening accuracy doubled these values (15.2% to 16.3%). The prevalence found by the two studies with the highest risk of bias (2.0% and 49.1%) were significantly divergent from the crude or adjusted values of the other studies.

**Fig 3:**
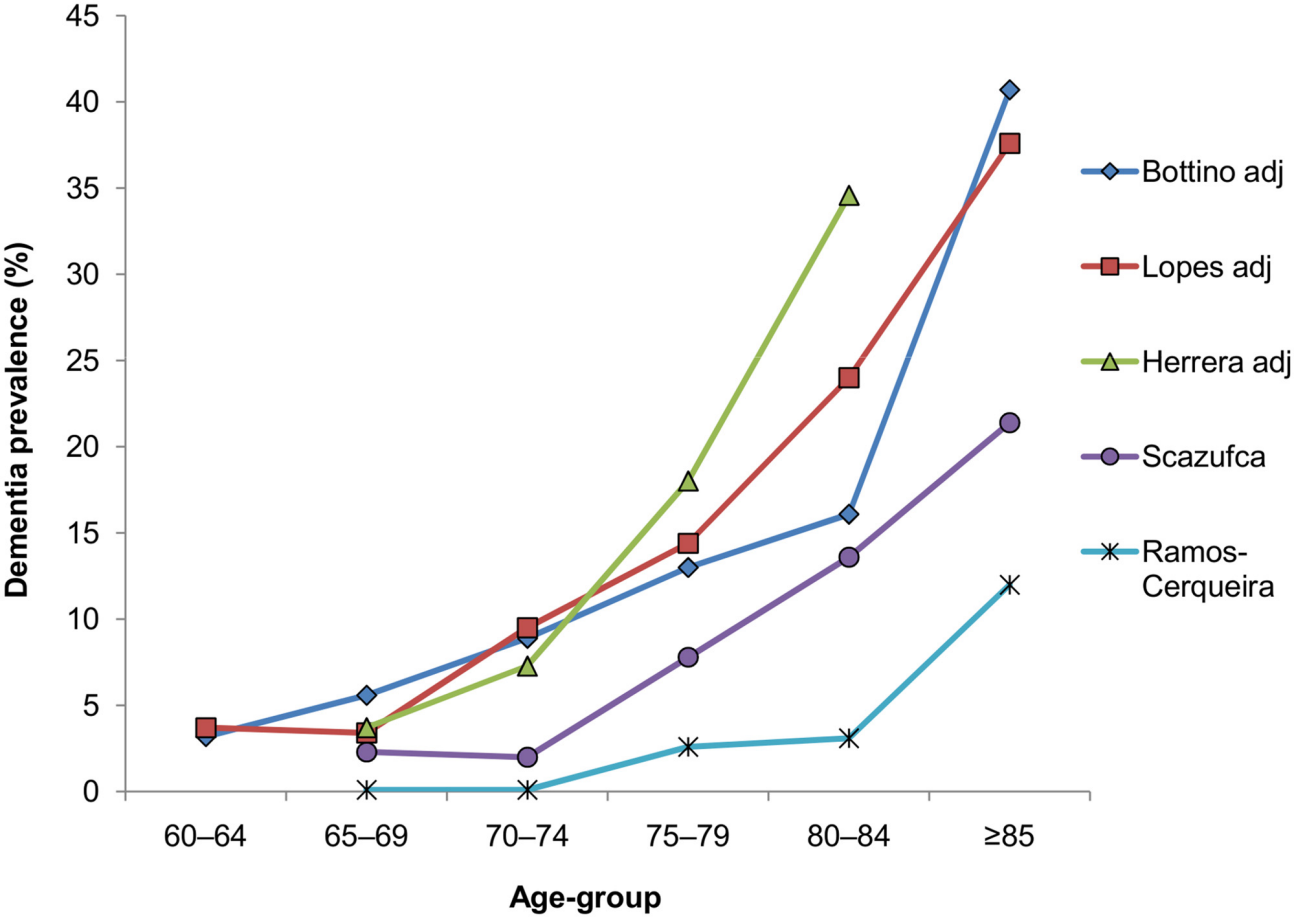
Age-specific prevalence of dementia among the studies selected for full analysis.*. *Magalhães: data not available. Herrera adjusted: prevalence in the group aged 85 years and older (88.7%) is not presented.

Fig 3 shows that among the six studies, the higher the age group, the higher the prevalence of dementia (S2 Table additionally presents non-adjusted prevalence from the studies of Bottino, Lopes and Herrera). However, differences *among surveys* regarding age-specific prevalence are relevant. The prevalence at 70–74 years of age found by Ramos-Cerqueira and Bottino were, respectively, 0.1% and 7.1%, whereas the adjusted prevalence at 80–84 years of age found by Bottino and Lopes—who used a similar methodology—were 16.1% and 24.0%. The differences *within each survey* regarding age variation were also considerable. While the prevalence at 65–69 years and 70–74 years did not vary in the surveys from Scazufca (2.3% and 2.0%) and Ramos-Cerqueira (0.1%), it doubled in the survey from Herrera (1.6% to 3.2%) and almost tripled in the survey from Lopes (2.2% to 6.3%). The prevalence found by Scazufca among those aged 85years and older (21.4%) was 10 times that found among those aged 70–74 years (2.0%), but was only three times higher in the study from Bottino (22.4% and 7.1%).

## Discussion

### Main findings

This is the first study, to our knowledge, to show that the risk of bias may strongly decrease the reliability of dementia prevalence estimates from surveys as input data for nationwide extrapolations in developing countries. The most accurate data regarding dementia prevalence for Latin America are derived from Brazil, where surveys have used highly reliable sampling techniques and appropriate screening and diagnostic procedures. For the purpose of a nationwide extrapolation, however, all the studies presented moderate or high risk of bias in at least two domains. These results are in agreement with those from Wu et al. [24] who recently estimated the dementia prevalence in mainland China, Taiwan and Hong Kong from local surveys. In this large study analyzing 76 surveys, they concluded that the heterogeneity of diagnostic criteria, age range, population size and sampling methods highly influenced the results.

Our study also shows that–in addition to the more common sources of bias—dementia surveys may be hindered by obstacles that are peculiar to developing countries. Most of them can hardly be recognized without a meticulous analysis of the methodology and results sections. An important problem is the high prevalence of low literacy among older people and its poorly known influence on cognitive tests. A linked problem is the unknown reliability of internationally accepted cognitive tests when applied in source populations in developing countries with less or no education. The causes of non-response may also be distinct from the patterns observed in developed countries, requiring different interpretations. Lastly, the accuracy of the examiners who actually applied the cognitive tests is almost never stated (if ever assessed).

### Risk of bias of the studies evaluated in this systematic review

#### Selection bias

In Brazil, the most significant obstacle to ascertain the nationwide dementia prevalence is the scarcity of surveys with adequate sampling methods. Among the 35 studies focusing on the prevalence of dementia, 15 were not population-based or addressed specific ethnic groups. Additionally, five population-based studies did not use random samples (or the whole community). Even among the studies with adequate sampling methods, none stratified the sample by age, thus compromising the representativeness of the small group aged 85 years and older, among which dementia is most common. The result is lack of precision which is illustrated in the prevalence and 95% confidence intervals found by Bottino et al. and by Lopes et al. among those aged 85 years and older, respectively, 22.5% (14.2, 30.7) and 29.6% (17.3, 41.9). Although the cluster sampling methodology of these two studies may have improved the precision of income-specific estimates, their samples no longer matched the actual proportions of each income classes in São Paulo, for which the observed prevalence should have been weighted. Finally, by not including the subjects who were living in nursing homes, dementia prevalence was certainly underestimated.

#### Outcome bias

Among the remaining 15 population-based random sample surveys (and surveys of the whole community), the risk of outcome bias was the most common limitation. This was mainly due to the utilization of poorly specified diagnosis criteria (such as “cognitive deterioration” or “cognitive deficit”) or non-validated methods for screening (such as the Brazil Old Age Schedule). An additional risk of outcome bias arose when the diagnosis of dementia relied solely on cognitive screening tests, such as the MMSE. Many studies did not perform clinical and functional evaluation, informant interviews, and laboratory tests or brain scans to exclude other causes of cognitive deficits. Furthermore, the role of the MMSE in low literacy populations—even as a screening test—has been much criticized [25].

Even the proper utilization of internationally accepted methods of diagnosis is not sufficient to avoid outcome bias; cultural and educational issues remain important challenges for accuracy [18]. Although some Brazilian studies utilized a version of CAMDEX translated and adapted to Portuguese [26], it was only in 2013 that a revised version, extensively cross-culturally adapted, became available [27]. These authors proved that the test is too difficult for illiterate individuals that comprised 51%, 17%, and 10% of the samples from Magalhães, Bottino, and Lopes. Furthermore, the cut-off used in the Brazilian surveys (79/80) was originally proposed for subjects with at least eight years of schooling [28], which was not the case for almost 2/3 of the samples by Bottino and by Lopes. In these two studies, however, higher rates of false positives were probably avoided by the evaluation of all possible dementia cases by experts (Lopes MA, personal communication). Regarding the 10/66 protocol, its (high) accuracy was demonstrated in a pilot study against a gold standard of mild-to-moderate dementia cases [18], which could raise concerns about the underestimation of prevalence by missing milder cases. However, a clinical validation study [29] further confirmed its accuracy also for mild cases. On the contrary, some concern has been raised that in some studies in Latin America the differential diagnosis of dementia may not have been distinguished sufficiently from other mental health conditions, thus increasing the risk of overestimating dementia prevalence [18].

In a broader view, the diagnostic criteria itself—DSM-IV, International Classification of Diseases, 10^th^ Revision (ICD 10), CAMDEX—may bring enough variability to impair any comparison between surveys. Erkinjuntti et al. [30] demonstrated that the prevalence of dementia in a large population (10,263 Canadian subjects aged 65 years and older) varied between 3.1% and 13.7% when respectively the ICD-10 and DSM-IV were utilized for the diagnosis. Surprisingly, when the diagnosis was based solely on clinical consensus, the prevalence was 20.9%, even higher than the adjusted values from the surveys of Bottino, Lopes, and Herrera. In the same line, Wu et al. [24] showed that the prevalence almost doubled when the DSM-IV criterion—and not the DSM-III—was utilized in China. On the other hand, Prince et al. [29] showed that the gain in specificity with the DSM-IV stricter criteria was largely offset by loss of sensitivity and underestimation of prevalence (one third of the mild cases in Cuba) when compared to the 10/66 protocol or clinical consensus.

One last issue in outcome bias is the uncertainty related to the level of expertise of the examiners. Their accuracy—if ever assessed—was seldom presented, and does not necessarily match the accuracy of the examiners involved in the original validation of the screening and diagnostic methods. The validation procedure itself, most of the time, is done under better, *quasi* artificial conditions: e.g., experts performing the tests at university hospitals.

#### Attrition bias

Systematic differences between groups in withdrawals proved to be a significant concern to internal validity in this review. In their studies Bottino and Lopes were not able to screen almost one third of the eligible subjects: they refused the evaluation. Refusal rates may be related to the health status of eligible subjects, but also to training and expertise of the interviewers to avoid selective participation. Both patterns would highly influence the results [31]. A similar proportion was not evaluated at their 2^nd^ phases although, in this case, the authors succeeded in comparing the missing subjects with those who were actually evaluated, and demonstrated that the groups were quite similar.

#### Statistical issues

The importance to back-weighting the prevalence by the screening accuracy was demonstrated by the studies of Bottino and Lopes, as their prevalence almost doubled with this procedure. Although the evaluation of a sample of the screen-negative subjects is included among the quality criteria proposed by Prince et al. [1], it is rarely done in two phase studies. In the same way, the clinical validation of one phase studies must be assured as this methodology assumes accuracies high enough to exempt the evaluation of a sample of negative subjects.

Another important statistical issue is the attempt of estimating the age-specific prevalence of dementia. The precision of these sub-group estimations was poor since the sample size is usually calculated for the whole group.

#### Population bias

Given its high internal validity, Scazufca’s survey results are generalizable to low income sectors of the Brazilian population; its use for nationwide extrapolations, however, would induce population bias. Indeed, selective survival of healthier subjects in this population could reduce the proportion of demented people [32] and may explain the lower prevalence they found. A higher mortality rate among those with dementia (early censoring of cases) is another possibility proposed by the authors themselves. The results from Magalhães, who evaluated a rural population, are also not generalizable.

### Risk of bias of other estimates for Brazil and Latin America

The studies we have selected in our article are different than those selected by other systematic reviews on dementia prevalence in Brazil and Latin America published recently. Since our aim was to estimate the nationwide prevalence of dementia in Brazil, we excluded follow-ups of cohort studies aimed at incidence, studies on dementia in specific ethnic groups or in a not randomly selected population and studies in which the diagnosis was based solely on screening tests.

Fagundes et al. [33] carried out a systematic review of surveys published from 1990 to 2010 and identified 112 articles, of which 11 consisted of population-based studies with cognitive tests, such as MMSE [34] to evaluate dementia and CFI [10]. Their meta-regression indicated that in low-quality studies the prevalence of dementia was overestimated, and large heterogeneity between studies was observed due to different age selection and socio-economic status. These limitations prohibited a meta-analysis on the best estimate of dementia in Brazil. In another systematic review, Burlá et al. [35] selected eight out of 703 studies and calculated a pooled prevalence of dementia by weighting the prevalence found in each study by the sample size. However, this pooling procedure was done regardless of random sampling and, thus included two surveys within specific ethnic groups.

On a broader perspective, Prince [36] projected the number of cases of dementia in developing countries in 2000, but assumed a global uniform prevalence rate of 3% disregarding, for example, differences in the age distribution of the older population. Wimo et al. [37], although taking into account age-specific differences in prevalence, assumed that they should be uniform among continents and used a review from Argentina [38] as the only data source for the Latin-America prevalence. Ferri et al. [39], through a consensus method supported by a systematic review, estimated the age-specific prevalence of dementia for each of the 14 WHO regions and also based the estimates for Latin-America on a single survey. The systematic review and meta-analysis recently accomplished by Prince et al. [1] included 11 studies carried out in Latin-America (only one back-weighting the prevalence by the screening accuracy). The Brazilian sources of data were only the surveys from Herrera, Scazufca and Bottino since the 2^nd^ phase study from Lopes et al. [21] had not been published yet. For the reasons we presented, their estimates of 8.5% prevalence of dementia (among those aged 60 years and older, age-standardized by the Western European population) must be interpreted with caution.

### A nationwide dementia estimate for Brazil

Notwithstanding the limitations discussed above, some conclusions may be drawn from the estimates currently available. The four studies with the lower risk of bias (Bottino et al., Lopes et al., Herrera et al. and Scazufca et al.) provided a very similar non-adjusted prevalence for subjects aged 65 years and older, ranging from 5.1% to 8.1%, which is very close to that found by Prince et al. [1] for Latin America. By adjusting the results according to the accuracy of the screening applied (in the two-phase studies with the lowest risk of bias), the prevalence almost doubled, and ranged from 15.2% to 16.3% for subjects aged 65 years and older, which—according to the criteria we have selected—are the best estimates currently available for Brazil.

Due to the heterogeneity of the population, an accurate estimate of dementia prevalence should include data from all Brazilian regions since most studies so far have been developed in the Southeast region. It would also be necessary to perform additional adjustments before carrying out a meta-analysis. The cluster sampling procedures from Bottino and Lopes created artificial populations with almost equal proportions of the three socio-economic classes, which does not correspond to the source population, and should be adjusted accordingly. The prevalence found by Scazufca could be included in the meta-analysis if weighted to the proportion of low income subjects aged 60 years and older.

The current study has some limitations. First, the selection in the systematic review was carried out by one author and this could lead to missing information or cause bias related to the application of inclusion and exclusion criteria. However, the total number of reports was not so large, and all the steps of the systematic review were double checked. Furthermore, due to the very specific evaluation of the methodology (e.g., if prevalence data was presented; if the sample was population-based) we believe that the chance of bias was minimized. A second limitation relates to the subjective classification of the magnitude of the risk of bias. Nevertheless, this step was done in agreement by the two authors, the reasons for the classification are fully presented in the results, and even a misclassification (between low/high risks) would not change our main conclusions. Finally, if more information were available—for example, the accuracy of the examiners who applied the tests—some of our findings might have been different.

This systematic review was able to identify common sources of bias that could be avoided by the implementation of well known procedures: a representative population based on random samples, including nursing home residents; stratification by age groups assuring the representativeness of those aged 85 and older; cluster sampling by income groups and further adjustment for the source population; adequate sample size for estimation of age-specific prevalences; baseline measurements for all participants recruited enabling the analysis of selective participation; use of internationally accepted criteria for diagnosis of dementia; and adjustment of the prevalence for the screening accuracy. However, the implementation of all these advisable procedures may not be feasible if budget is restricted.

In conclusion, notwithstanding the expected increase in the burden of dementia and the urgent need to plan public health policies, this study suggests that accurate data on its prevalence may be lacking in developing countries. We showed that moderate to high risk of selection, attrition, outcome and population bias, as well as statistical issues are strong limitations for the generalizability of current data in Brazil. Although many estimates have already been calculated for Brazil and Latin America, we demonstrated that all of them were based on a prevalence that should have been adjusted—or not used at all. Similar evaluations regarding other developing countries and global regions are needed in order to verify the scope of these limitations. To provide useful data for nationwide estimations further surveys should strictly follow the existing recommendations to avoid bias and might benefit from the approach that we developed.

## Supporting Information

S1 Protocol Protocol for the systematic review. (PDF)

S1 PRISMA Checklist PRISMA Checklist. (PDF)

S1 File Full search strategy. (PDF)

S2 File Articles excluded at the eligibility assessment. (PDF)

S3 File Checklist to assess the risk of bias. (PDF)

S1 Table Prevalence of dementia on the six surveys fully analyzed. (PDF)

S2 Table Age-specific prevalences of dementia. (PDF)
