# Supplementary material for: Reliability of Nationwide Prevalence Estimates of Dementia: A Critical Appraisal Based on Brazilian Surveys
Source: PLoS One. 2015 Jul 1;10(7):e0131979. doi: 10.1371/journal.pone.0131979 (PMC4488471; doi:10.1371/journal.pone.0131979)
Supplement: S1 Protocol — (PDF) [file pone.0131979.s001.pdf]

Appendix S1: Protocol for the systematic review

UNIVERSITY of York
Centre for Reviews and Dissemination

NHS
National Institute for Health Research

PROSPERO International prospective register of systematic reviews

Review title and timescale

1 Review title
Give the working title of the review. This must be in English. Ideally it should state succinctly the interventions or exposures being reviewed and the associated health or social problem being addressed in the review.
Dementia prevalence in Brazil

2 Original language title
For reviews in languages other than English, this field should be used to enter the title in the language of the review. This will be displayed together with the English language title.

3 Anticipated or actual start date
Give the date when the systematic review commenced, or is expected to commence.
05/03/2014

4 Anticipated completion date
Give the date by which the review is expected to be completed.
04/04/2014

5 Stage of review at time of this submission
Indicate the stage of progress of the review by ticking the relevant boxes. Reviews that have progressed beyond the point of completing data extraction at the time of initial registration are not eligible for inclusion in PROSPERO. This field should be updated when any amendments are made to a published record.

The review has not yet started ×

Review stage	Started	Completed
Preliminary searches	Yes	No
Piloting of the study selection process	Yes	No
Formal screening of search results against eligibility criteria	No	No
Data extraction	No	No
Risk of bias (quality) assessment	No	No
Data analysis	No	No

Provide any other relevant information about the stage of the review here.
Research funded by CAPES (Brazil).

Review team details

6 Named contact
The named contact acts as the guarantor for the accuracy of the information presented in the register record.
Flavio Chaimowicz

7 Named contact email
Enter the electronic mail address of the named contact.
flaviochz@gmail.com

8 Named contact address
Enter the full postal address for the named contact.
Rua Agripa de Vasconcelos, 190. 30210-030 Belo Horizonte MG Brazil

9 Named contact phone number
Enter the telephone number for the named contact, including international dialing code.
+553132270967

10 Organisational affiliation of the review
Full title of the organisational affiliations for this review, and website address if available. This field may be completed as 'None' if the review is not affiliated to any organisation.

Erasmus MC Department of Public Health, Rotterdam (The Netherlands); Internal Medicine Department, Faculty of Medicine, Federal University of Minas Gerais (Brazil)

Website address:
http://www.eur.nl/english/; http://www.ufmg.br/

11 Review team members and their organisational affiliations
Give the title, first name and last name of all members of the team working directly on the review. Give the organisational affiliations of each member of the review team.

Title	First name	Last name	Affiliation
Dr	Flavio	Chaimowicz	Internal Medicine Department, Faculty of Medicine, Federal University of Minas Gerais (Brazil)
Professor	Alex	Burdorf	Erasmus MC Department of Public Health

12 Funding sources/sponsors
Give details of the individuals, organizations, groups or other legal entities who take responsibility for initiating, managing, sponsoring and/or financing the review. Any unique identification numbers assigned to the review by the individuals or bodies listed should be included.
Partially funded by a CAPES scholarship (Brazil) # 5416-13-2

13 Conflicts of interest
List any conditions that could lead to actual or perceived undue influence on judgements concerning the main topic investigated in the review.
Are there any actual or potential conflicts of interest?
None known

14 Collaborators
Give the name, affiliation and role of any individuals or organisations who are working on the review but who are not listed as review team members.

Title	First name	Last name	Organisation details

Review methods

15 Review question(s)
State the question(s) to be addressed / review objectives. Please complete a separate box for each question.
The aim of this systematic review is to estimate the prevalence of dementia in Brazil.

16 Searches
Give details of the sources to be searched, and any restrictions (e.g. language or publication period). The full search strategy is not required, but may be supplied as a link or attachment.
We will search Pubmed, Science Direct, Lilacs, Scielo and the Brazilian thesis database. The search strategy will be: (dem?n?ia OR Alzheimer*) AND (preval?nc* OR epidemiolog*) AND (Bra?il)

17 URL to search strategy
If you have one, give the link to your search strategy here. Alternatively you can e-mail this to PROSPERO and we will store and link to it.
http://www.crd.york.ac.uk/PROSPEROFILES/8815_STRATEGY_20140204.pdf

I give permission for this file to be made publicly available
No

18 Condition or domain being studied
Give a short description of the disease, condition or healthcare domain being studied. This could include health and wellbeing outcomes.
Dementia in old age, including Alzheimer's disease

19 Participants/population

Give summary criteria for the participants or populations being studied by the review. The preferred format includes details of both inclusion and exclusion criteria.

Elderly people (60+ years old) living in the comunity

20 Intervention(s), exposure(s)

Give full and clear descriptions of the nature of the interventions or the exposures to be reviewed

This systematic review and meta-analysis will estimate the prevalence of dementia among people aged 60 years and over based on prevalence studies that diagnosed the disease according to DSM-IV, ICD-10 or similar criteria recommended by the Brazilian Academy of Neurology [Nitrini et al., 2005]. Exclusion criteria are detailed at the item 22

21 Comparator(s)/control

Where relevant, give details of the alternatives against which the main subject/topic of the review will be compared (e.g. another intervention or a non-exposed control group).

We will compare the prevalence among gender and age groups.

22 Types of study to be included initially

Give details of the study designs to be included in the review. If there are no restrictions on the types of study design eligible for inclusion, this should be stated.

We will evaluate population-based studies which data was collected in the last 30 years. Exclusion criteria: Studies of prevalence from the follow-up phase of on an incidence cohort; out-of-date population registers (not the last census available); specific ethnic, income or older age groups; ascertainment based on help-seeking and/or receipt of dementia care services; studies where 'dementia' was diagnosed purely on the basis of cognitive impairment or non-validated tests; one phase studies; two phase studies in which screening procedures were inadequate; two phase studies whose methodology was not properly applied; studies of the prevalence of Alzheimer's disease or other subtypes; significant differences between responders and non-responders.

23 Context

Give summary details of the setting and other relevant characteristics which help define the inclusion or exclusion criteria.

We will include population based studies. We will exclude studies related to nursing home or residential care populations, primary or secondary care attendees or other unrepresentative service-user populations.

24 Primary outcome(s)

Give the most important outcomes.

Prevalence of dementia

Give information on timing and effect measures, as appropriate.

25 Secondary outcomes

List any additional outcomes that will be addressed. If there are no secondary outcomes enter None.

Prevalence of dementia by gender and age groups. Prevalence of sub-types of dementia (e.g. Alzheimer's disease, vascular dementia).

Give information on timing and effect measures, as appropriate.

26 Data extraction, (selection and coding)

Give the procedure for selecting studies for the review and extracting data, including the number of researchers involved and how discrepancies will be resolved. List the data to be extracted.

Titles, abstracts and key-words of studies retrieved using the search strategy and those from additional sources will be screened independently by two review authors to identify studies that potentially meet the inclusion criteria outlined above. The full text of these potentially eligible studies will be retrieved and independently assessed for eligibility by two review team members. Any disagreement between them over the eligibility of particular studies will be resolved through discussion with a third reviewer. A standardized, pre-piloted form will be used to extract data from the included studies for assessment of study quality and evidence synthesis. Extracted information will include: study setting; sampling frame and design, study population and participant demographics and characteristics; study methodology; response rates in the 1st (screening) and 2nd (clinical evaluation) phases; description of refusers provided, methods utilized for the screening and clinical diagnosis of dementia, overall prevalence of dementia and prevalence by gender, age-group and type, presentation of results (confidence intervals provided and subgroup

analysis by age and sex presented) information for assessment of the risk of bias. Two review authors will extract data independently, discrepancies will be identified and resolved through discussion (with a third author where necessary). Missing data will be requested from study authors.

27 Risk of bias (quality) assessment

State whether and how risk of bias will be assessed, how the quality of individual studies will be assessed, and whether and how this will influence the planned synthesis.

We will consider the following characteristics: Population source: General population; not limited to specific groups (e.g. "lower income" or specific ethnic groups). Not based on nursing home or residential care populations, primary care attendees or other unrepresentative service-user populations, nor based on help-seeking and/or receipt of dementia care services. Sampling frame unbiased. Census data based, using the most up-to-date census data available. Sampling design: random sample or the whole population, preferably stratified by age-groups. Sample size =500 (preferably =1500) or =300 for each age-group‡. At least two phases: screening with good sensitivity and specificity and detailed clinical evaluation. Prevalence adjusted for screening accuracy. Not two phase studies in which screening procedures were inadequate or two phase studies whose methodology was not properly applied. Response rate = 60% in both phases. Refusers described, and with similar characteristics to non refusers. Reliable and valid outcome. Dementia diagnosed by worldwide standard criteria: ICD-10 or DSM-IV. Outcome measure unbiased: negative screens assessed. Preferably inter/intraobserver reliability assessed. Scope of diagnosis: Multidimensional assessment, based on cognitive, functional and clinical evaluations, and also interview with an informant. Not ascertainment studies where 'dementia' is diagnosed purely on the basis of cognitive impairment.

28 Strategy for data synthesis

Give the planned general approach to be used, for example whether the data to be used will be aggregate or at the level of individual participants, and whether a quantitative or narrative (descriptive) synthesis is planned. Where appropriate a brief outline of analytic approach should be given.

We will provide a narrative synthesis of the findings from the included studies and describe the main strengths and risk of bias of each one. We will estimate the overall and age/gender specific prevalence (and 95% CI) through a meta-analysis. Heterogeneity between the studies in effect measures will be assessed, as well as evidence of publication bias

29 Analysis of subgroups or subsets

Give any planned exploration of subgroups or subsets within the review. 'None planned' is a valid response if no subgroup analyses are planned.

We plan to estimate the prevalence by gender, age-group and type of dementia.

Review general information

30 Type of review

Select the type of review from the drop down list.

Epidemiologic

31 Language

Select the language(s) in which the review is being written and will be made available, from the drop down list. Use the control key to select more than one language.

English

Will a summary/abstract be made available in English?

Yes

32 Country

Select the country in which the review is being carried out from the drop down list. For multi-national collaborations select all the countries involved. Use the control key to select more than one country.

Netherlands

33 Other registration details

List places where the systematic review title or protocol is registered (such as with he Campbell Collaboration, or The Joanna Briggs Institute). The name of the organisation and any unique identification number assigned to the review by that organization should be included.

The research project is registered at NIHES-Erasmus MC, Erasmus University, Rotterdam.

34 Reference and/or URL for published protocol

Give the citation for the published protocol, if there is one.
Give the link to the published protocol, if there is one. This may be to an external site or to a protocol deposited with CRD in pdf format.

I give permission for this file to be made publicly available
No

35 Dissemination plans
Give brief details of plans for communicating essential messages from the review to the appropriate audiences.
The results will be freely available at Plos One, if accepted for publication.

Do you intend to publish the review on completion?
Yes

36 Keywords
Give words or phrases that best describe the review. (One word per box, create a new box for each term)
Dementia

Prevalence

Brazil

Systematic review

Meta-analysis

37 Details of any existing review of the same topic by the same authors
Give details of earlier versions of the systematic review if an update of an existing review is being registered, including full bibliographic reference if possible.

38 Current review status
Review status should be updated when the review is completed and when it is published.
Ongoing

39 Any additional information
Provide any further information the review team consider relevant to the registration of the review.

40 Details of final report/publication(s)
This field should be left empty until details of the completed review are available.
Give the full citation for the final report or publication of the systematic review.
Give the URL where available.
