# Supplementary material for: Reliability of Nationwide Prevalence Estimates of Dementia: A Critical Appraisal Based on Brazilian Surveys
Source: PLoS One. 2015 Jul 1;10(7):e0131979. doi: 10.1371/journal.pone.0131979 (PMC4488471; doi:10.1371/journal.pone.0131979)
Supplement: S1 File — (PDF) [file pone.0131979.s003.pdf]

Appendix S3: Full search strategy

Databases	Search strategy	N
**Medline**	("dementia"[All Fields] OR "Alzheimer"[All Fields]) AND ("prevalence"[All Fields] OR "epidemiology"[All Fields]) AND ("Brazil"[All Fields] OR "Brasil"[All Fields])	194
**LILACS**	demencia OR demência OR dementia OR Alzheimer [Palavras] and prevalencia OR prevalência OR prevalence OR epidemiologia OR epidemiology [Palavras] and Brasil OR Brazil [Palavras]	106
**SciELO**	demencia OR demência OR dementia OR Alzheimer [All indexes] and prevalencia OR prevalência OR prevalence OR epidemiologia OR epidemiology [All indexes] and Brasil OR Brazil [All indexes]	62
**BDTD***	Demência and prevalência	7

*Brazilian Thesis Databases
