# Supplementary material for: Reliability of Nationwide Prevalence Estimates of Dementia: A Critical Appraisal Based on Brazilian Surveys
Source: PLoS One. 2015 Jul 1;10(7):e0131979. doi: 10.1371/journal.pone.0131979 (PMC4488471; doi:10.1371/journal.pone.0131979)
Supplement: S2 File — (PDF) [file pone.0131979.s004.pdf]

Appendix S4: Articles excluded at the eligibility assessment

**Studies evaluating specific ethnic groups**		
**1**	192	Meguro K, Meguro M, Caramelli P, Ishizaki J, Ambo H et al. (2001). Elderly Japanese emigrants to Brazil before World War II: II. Prevalence of senile dementia. Int J Geriatr Psychiatry 16(8):775-779.
**2**	357	Yamada T, Kadekaru H, Matsumoto S, Inada H, Tanabe M et al. (2002) Prevalence of dementia in the older Japanese-Brazilian population. Psychiatry Clin Neurosci 56(1):71-75.
**3**	376	Meguro K, Meguro M, Caramelli P, Ishizaki J, Ambo H et al. (2001). An environmental change does not affect dementia prevalence but affects depressive state and physical activity: a trans-cultural study of Japanese elderly subjects and Japanese elderly immigrants in Brazil. Psychogeriatrics 1: 201–208.
**4**	383	Bandeira-Lopes D (2012) [Prevalence of cognitive and functional disorders among older people living at Kalunga quilombola community]. M. Sc. Thesis, Federal University of Goias. Available: http://www.odonto.ufg.br/uploads/133/original_danielly.pdf Accessed 27 November 2014. Portuguese.

**Additional reports from other main studies**			**Main**
**5**	22	Benseñor IM, Lotufo PA, Menezes PR, Scazufca M (2010) Subclinical hyperthyroidism and dementia: the Sao Paulo Ageing & Health Study (SPAH). BMC Public Health 10:298.	Scazufca 2007
**6**	121	Folquitto JC; Marques RCG; Tatsch MF; Bottino CMC (2013) Correlation between neuropsychiatric symptoms and caregiver burden in a population-based sample from São Paulo, Brazil: a preliminary report. Dement Neuropsychol 7(3):258-262.	Bottino 2008
**7**	144	Herrera Junior E, Caramelli P, Nitrini R (1998) Population epidemiologic study of dementia in Catanduva city: state of Sao Paulo, Brazil. Rev Psiquiatr Clin (São Paulo) 25(2):70-73.	Herrera 2002
**8**	174	Lopes MA, Hototian SR, Bustamante SE, Azevedo D, Tatsch M (2007) Prevalence of cognitive and functional impairment in a community sample in Ribeirão Preto, Brazil. Int J Geriatr Psychiatry 22(8):770-776.	Lopes 2012
**9**	225	Oliveira SFD, Duarte YAO, Lebrão ML, Laurenti R (2007) [Demands Reported and Help Received among Elderly People with Cognitive Decline at Sao Paulo Municipality]. Saude Soc 16(1):81-89. Portuguese.	Lebrão 2002
**10**	261	Santos IS, Scazufca M, Lotufo PA, Menezes PR, Benseñor IM (2012) Anemia and dementia among the elderly: the São Paulo Ageing & Health Study. Int Psychogeriatr 24(1):74-81.	Scazufca 2007
**11**	267	Scazufca M, Almeida OP, Menezes PR (2010) The role of literacy, occupation and income in dementia prevention: the São Paulo Ageing & Health Study. Int Psychogeriatr 22(8):1209-1215.	Scazufca 2007
**12**	268	Scazufca M, Almeida OP, Vallada HP, Tasse WA, Menezes PR (2009) Limitations of the Mini-Mental State Examination for screening dementia in a community with low socioeconomic status: results from the Sao Paulo Ageing & Health Study. Eur Arch Psychiatry Clin Neurosci 259(1):8-15.	Scazufca 2007
**13**	272	Scazufca M, Menezes PR, Araya R, Di Rienzo VD, Almeida OP et al (2008) Risk factors across the life course and dementia in a Brazilian population: results from the Sao Paulo Ageing & Health Study (SPAH). Int J Epidemiol 37(4):879-890.	Scazufca 2007
**14**	313	Tatsch MF, Bottino CM, Azevedo D, Hototian SR, Moscoso MA et al. (2006) Neuropsychiatric symptoms in Alzheimer disease and cognitively impaired, nondemented elderly from a community-based sample in Brazil: prevalence and relationship with dementia severity. Am J Geriatr Psychiatry 14(5):438-445.	Bottino 2008
**15**	337	Veras RP, Coutinho E (1994) [Prevalence of organic brain syndrome in an elderly population in a metropolitan area of the southeastern region of Brazil]. Rev Saude Publica 28(1):26-37. Portuguese.	Veras 1991
**16**	364	Ribeiro PCC (2012) [Prevalence of dementia and variables associated to cognitive decline among elderly clients from a private healthcare plan]. Ph. D. Thesis, University of Rio de Janeiro. Available: http://www.ims.uerj.br/downloads/dissertacoes/Pricila%20Cristina%20Correa%20Ribeiro%20(Cont.)0001.pdf Accessed 27 November 2014. Portuguese.	Ribeiro 2013
**17**	365	Lopes MA (2006) [Epidemiological survey of prevalence of dementia in Ribeirão Preto]. Ph. D. Thesis, São Paulo University. Available: http://www.teses.usp.br/teses/disponiveis/5/5142/tde-18042007-110300/pt-br.php Accessed 27 November 2014. Portuguese.	Lopes 2007
**18**	368	Santos I (2009) [Anemia prevalence in older subjects, causes of persistence or recurrence and its relation with dementia]. Ph. D. Thesis, São Paulo University. Available: http://www.teses.usp.br/teses/disponiveis/5/5159/tde-05022010-164250/pt-br.php Accessed 27 November 2014. Portuguese.	Scazufca 2007
**19**	369	Tatsch MF (2006) [Behavioral and psychological symptoms of dementia in a community dwelling São Paulo-Brazilian population: prevalence, relationship with dementia severity and with caregiver burden]. Ph. D. Thesis, São Paulo University. Available: http://www.teses.usp.br/teses/disponiveis/5/5142/tde-28042006-135736/pt-br.php Accessed 27 November 2014. Portuguese.	Bottino 2008

**20**	377	Caramelli P, Barbosa MT, Beato RG, et al. (2013) Variables associated with cognitive impairment and dementia in a low-educated cohort aged 75+ years: The PIETÀ study. Dement Neuropsychol 7(Suppl 1):15-16.	Caramelli 2011.
**21**	378	Caramelli P, Teixeira AL, Barbosa MT, Santos AP, Pellizzaro M et al. (2009) Prevalence of cognitive impairment and dementia in a cohort of oldest old in Brazil: The Pietà study. Alzheimer's & Dementia 5(4):P391-2.	Caramelli 2011.
**22**	390	Hototian S, Lopes MA, Bustamante SEZ, Azevedo D, Tatsch M, et al. (2005) Identification of dementia suspects subjects in three districts of São Paulo city, Brazil. Int Psychogeriatr 17:271-272.	Bottino 2008
**23**	391	Hototian S, Lopes MA, Azevedo D, Tatsch M, Bazzarella MC, et al. (2008) Prevalence of cognitive and functional impairment in a community sample from São Paulo, Brazil. Dement Geriatr Cogn Disord 25(2):135-143.	Bottino 2008
**24**	392	Hototian, SR ; Lopes, MA ; Bustamante, SEZ ; Azevedo, D ; Tatsch, MF, et al. (2004) Dementia prevalence study in a community sample of Sao Paulo, Brazil. Neurobiol Aging 25: S479.	Bottino 2008

**Studies not focusing on dementia prevalence**		
**25**	15	Baldivia B, Brucki SM, Batistela S, Esper JC, Augusto CD et al. (2011) Dementia in Parkinson's disease: a Brazilian sample. Arq Neuropsiquiatr 69(5):733-738.
**26**	131	Fujihara S, Brucki SM, Rocha MS, Carvalho AA, Piccolo AC (2004) Prevalence of presenile dementia in a tertiary outpatient clinic. Arq Neuropsiquiatr 62(3A):592-595.
**27**	306	Takada LT, Caramelli P, Radanovic M, Anghinah R, Hartmann AP et al. (2003) Prevalence of potentially reversible dementias in a dementia outpatient clinic of a tertiary university-affiliated hospital in Brazil. Arq Neuropsiquiatr 61(4):925-929.
**28**	384	Vega UM, Marinho V, Engelhardt E, Laks J (2007) Neuropsychiatric symptoms in dementias: preliminary report of a prospective outpatient evaluation in Brazil. Arq Neuropsiquiatr 65:498-502.
**29**	385	Godinho C, Gorczevski I, Heisler A, Cerveira MO, Chaves ML (2010) Clinical and demographic characteristics of elderly patients with dementia assisted at an outpatient clinic in Southern Brazil. Dement Neuropsychol 4:42-46.
**30**	388	Tascone L, Marques RC, Pereira EC, Bottino CMC (2008) Characteristics of patients assisted at an ambulatory of dementia from a University Hospital. Arq Neuropsiquiatr 66:631-635.

**Not population based**		
**31**	23	Bezerra AB, Coutinho ES, Barca ML, Engedal K, Engelhardt E et al. (2012) School attainment in childhood is an independent risk factor of dementia in late life: results from a Brazilian sample. Int Psychogeriatr 24(1):55-61.
**32**	66	Ribeiro PCC, Lopes CS, Lourenço RA (2013) Prevalence of dementia in elderly clients of a private health care plan: a study of the FIBRA-RJ, Brazil. Dement Geriatr Cogn Disord 35(1-2):77-86.
**33**	197	Miranda Lde P, Silveira MF, Oliveira TL, Alves SF, Júnior HM et al. (2012) Cognitive impairment, the Mini-Mental State Examination and socio-demographic and dental variables in the elderly in Brazil. Gerodontology 29(2):e34-40.
**34**	224	Oliveira KCV, Barros ALS, Souza GFM (2007) Clinical cognitive profile of the elderly seen at Hospital Geral de Areias, Recife, Pernambuco, Brazil. Rev Ciênc Méd (Campinas) 16(3):151-159.
**35**	295	Sousa RG, Lovisi GM (2007) The appraisal of cognitive impairment in homeless people aged 65 years or over admitted to a public hostel. Rev Psiquiatr Clín (São Paulo) 34(5):205-209.
**36**	343	Viana GSB, Rouquayrol MZ (1988) Dementia cases in Fortaleza, Brazil: I. Data of records of a psychiatric hospital in the period 1978-1985. Rev ABP-APAL 10(3):79-82.
**37**	345	Vieira RT, Barros NM, Caixeta L, Machado S, Silva AC, Nardi AE (2013) Clinical diagnosis of 80 cases of dementia in a university hospital. J Bras Psiquiatr 62(2):139-143.
**38**	363	Canineu PR (2001) [Dementia prevalence among older patients (>= 60 years) living at "Dr. Candido Ferreira" healthcare center from Campinas Municipality]. Ph. D. Thesis, Unicamp. Available: http://www.bibliotecadigital.unicamp.br/document/?code=vtls000232368 Accessed 27 November 2014. Portuguese.
**39**	370	Almeida OP, Forlenza OV, Lima NK, Bigliani V, Arcuri SM (1997) Psychiatric morbidity among the elderly in a primary care setting--report from a survey in São Paulo, Brazil. Int J Geriatr Psychiatry 12(7):728-736.
**40**	386	Laks J, Marinho V (1997) [The geriatric psychiatry unit of Instituto de Psiquiatria da UFRJ: goals, description case and profile care]. J Bras Psiquiatr 46(10): 543-546. Portuguese.
**41**	387	Silva DW, Damasceno BP (2002) [Dementia among patients from Hospital das Clínicas da Unicamp]. Arq Neuropsiquiatr 60:996-999. Portuguese.

Not random sample		
**42**	45	Caramelli P, Barbosa MT, Sakurai E, Dos Santos EL, Beato RG et al. (2011) The Pietà study: epidemiological investigation on successful brain aging in Caeté (MG), Brazil. Methods and baseline cohort characteristics. Arq Neuropsiquiatr 69(4):579-584.
**43**	161	Laks J, Batista EM, Guilherme ER, Contino AL, Faria ME et al. (2005) Prevalence of cognitive and functional impairment in community-dwelling elderly: importance of evaluating activities of daily living. Arq Neuropsiquiatr 63(2A):207-212.
**44**	165	Laks J, Batista EMR, Guilherme ERL, Contino ALB, Faria MEV et al. (2003) [Mini-mental state examination in community-dwelling elderly: preliminary data from Santo Antônio de Pádua, Rio de Janeiro, Brazil]. Arq Neuropsiquiatr 61(3B):782-785. Portuguese.
**45**	178	Machado JC, Ribeiro RCL, Cotta RMM, Leal PFG (2011) [Cognitive decline of aged and its association with epidemiological factors in the city of Viçosa, Minas Gerais]. Rev Bras Geriatr Gerontol 14(1):109-121. Portuguese.
**46**	284	Silva EF, Paniz VMV, Laste G, Torres ILS (2013) [The prevalence of morbidity and symptoms among the elderly: a comparative study between rural and urban areas]. Ciênc Saúde Coletiva 18(4):1029-1040. Portuguese.

Diagnosis based on screening tests		
**47**	20	Benedetti TR, Borges LJ, Petroski EL, Gonçalves LH (2008) [Physical activity and mental health status among elderly people]. Rev Saúde Pública 42(2):302-307. Portuguese.
**48**	39	Lebrão ML, Laurenti R (2005) Health, well-being and aging: the SABE study in São Paulo, Brazil. Rev Bras Epidemiol 8:127-141.
**49**	339	Veras RP, Coutinho ES (1991) Prevalence of depression and organic cerebral syndrome in the elderly population, Brazil. Rev Saúde Pública 25(3):209-217.
**50**	341	Viana GS, Rouquayrol MZ, Bruin VM, Albuquerque JJ (1991). Use of the Information, Memory and Concentration (IMC) Test in the epidemiological study of senile dementia in Fortaleza,Ceará (Brazil). Cad Saude Publica 7(3):396-408.
**51**	367	Nascimento NMR (2008) [Comparative study of cognitive decline prevalence among two elderly groups]. M. Sc. Thesis, PUCRS. Available: http://repositorio.pucrs.br/dspace/handle/10923/3609#preview Accessed 27 November 2014. Portuguese.
**52**	372	Ramos LR, Rosa TEC, Oliveira ZM, Medina MCG, Santos FRG (1993). Profile of the elderly residents in Sao Paulo, Brazil: Results from a household survey. Rev Saúde Pública 27: 87-94.
**53**	373	Ramos LR, Toniolo Neto J, Cendoroglo MS, Garcia JT, Najas MS et al. (1998) Two-year follow-up study of elderly residents in S. Paulo, Brazil: methodology and preliminary results. Rev Saúde Pública 32:397-407.
**54**	375	Coelho Filho JM, Ramos LR (1999). [Epidemiology of ageing in Northeastern Brazil: results of a household survey]. Rev Saúde Pública 33(5): 445-453. Portuguese.
**55**	380	Valle EA, Castro-Costa E, Firmo JOA, Uchôa E, Lima-Costa MF (2009). [A population-based study on factors associated with performance on the Mini-Mental State Examination in the elderly: the Bambuí Study]. Cad Saúde Pública 25:918-926. Portuguese.

Follow up of cohorts		
**56**	62	Chaves ML, Camozzato AL, Godinho C, Piazenski I, Kaye J (2009) Incidence of mild cognitive impairment and Alzheimer disease in Southern Brazil. J Geriatr Psychiatry Neurol 22(3):181-187.
**57**	374	Ramos LR, Simões E, Albert MS (2001). Dependency on daily living and cognitive impairment strongly predicted mortality among urban elderly residents in Brazil: A two-year follow-up. J Am Geriat Soc 49:1168-1175.
**58**	379	Montaño MBMM, Ramos LR (2005) Validity of the Portuguese version of Clinical Dementia Rating. Rev Saúde Pública 39:912–917.
