# Supplementary material for: Reliability of Nationwide Prevalence Estimates of Dementia: A Critical Appraisal Based on Brazilian Surveys
Source: PLoS One. 2015 Jul 1;10(7):e0131979. doi: 10.1371/journal.pone.0131979 (PMC4488471; doi:10.1371/journal.pone.0131979)
Supplement: S3 File — (PDF) [file pone.0131979.s005.pdf]

Appendix S5: Checklist to assess the risk of bias

**General data**						
1	ID					
2	Citation					
3	Designed to determine dementia prevalence?					
4	Setting, locations					
5	Periods of recruitment					
6	Period of data collection					
**Sample characteristics**						
7	Size					
8	Age (mean)					
9	Age groups					
10	Income groups					
11	Literacy					
**INTERNAL VALIDITY ISSUES**						
**SELECTION BIAS**	**probability**		**magnitude**		**importance**	
12	Inclusion criteria clear?					
13	Exclusion criteria clear?					
14	Census based sampling frame?					
15	Source population well defined?					
16	Stratification by age groups?					
17	Stratification by income groups?					
18	Randomization s procedure adequate?					
19	Baseline measurements obtained for all participants recruited (immediate drop-outs)?					
20	Detection bias: any corrections made regarding those who recently moved to NH?					
**Results**						
21	Total prevalence					
22	Gender					
23	Age groups					
24	Income level					
25	Literacy					
**ATTRITION BIAS**	**probability**		**magnitude**		**importance**	
	Reported numbers of individuals					
26	- Potentially/examined/confirmed eligible?					
27	- Included in the study?					
28	- Completing follow-up?					
29	- Analyzed?					
30	Missing data adequately described?					
31	Proportion of non-respondents described?					
32	Response rate in the screening:					
33	Response rate in the clinical evaluation:					
34	Non-responders similar to responders?					
35	Results likely to be affected by losses between screening and clinical evaluation?					
36	Results likely to be affected by exclusions from analysis (e.g. missing values of birth date)?					

Continued on the next page

Continuation

OUTCOME BIAS		probability		magnitude		importance	
37	Outcome measure appropriate?						
	Diagnostic assessment						
38	- Multidomain cognitive testing?						
39	- Disability assessment?						
40	- Clinical interview?						
41	- Informant interview?						
	Outcome measure bias						
42	- Two independent assessors?						
43	- Inter-observer reliability assessed?						
44	- Intra-observer reliability assessed?						
	Performance bias						
45	- Assessors blinded for age, income, literacy, and clinical characteristics?						
46	- Assessors blinded for the screening result?						
	Objective method of measuring outcome						
47	- Validated instruments for screening?						
48	- Validated instruments for diagnostic?						
49	Outcome measured accurately: by professionals adequately trained/certified?						
50	Missing data adequately addressed?						
51	Reporting bias: selective outcome reporting?						
**STATISTICAL ANALYSIS**		**issues**		**magnitude**		**importance**	
52	Screening presented good sensitivity?						
53	Screening presented good specificity?						
54	Sample of screen negatives evaluated?						
55	Prevalence adjusted for screening accuracy?						
**EXTERNAL VALIDITY ISSUES**							
**POPULATION BIAS**		**probability**		**magnitude**		**importance**	
56	Sociodemographic variables detailed?						
57	Subjects representative of larger population?						
58	Sample size calculation adequate?						
59	Sample size ≥1500?						
60	Sample size ≥300 for each age-group?						
**Additional observations**							
61							
