# Supplementary material for: Reliability of Nationwide Prevalence Estimates of Dementia: A Critical Appraisal Based on Brazilian Surveys
Source: PLoS One. 2015 Jul 1;10(7):e0131979. doi: 10.1371/journal.pone.0131979 (PMC4488471; doi:10.1371/journal.pone.0131979)
Supplement: S1 Table — (PDF) [file pone.0131979.s006.pdf]

Table S1: Prevalence of dementia on the six surveys fully analyzed

First author	Age group	Prevalence (%)	95% CI
Bottino	65+	8.3	6.7-9.9
Bottino adjusted	65+	16.2	13.7-18.7
Lopes	65+	7.2	5.5-8.9
Lopes adjusted	65+	15.2	12.4-18.8
Herrera	65+	7.1	5.9-8.4
Herrera adjusted	65+	16.3	14.5-18.0
Scazufca	65+	5.1	4.1-6.0
Ramos-Cerqueira	65+	2.0	1.4-2.6
Magalhães	60+	49.1	46.0-52.3
Bottino	60+	6.8	5.5-8.1
Bottino adjusted	60+	12.9	10.9-14.9
Lopes	60+	5.9	4.5-7.3
Lopes adjusted	60+	12.5	11.0- 14.0
