# Supplementary material for: Reliability of Nationwide Prevalence Estimates of Dementia: A Critical Appraisal Based on Brazilian Surveys
Source: PLoS One. 2015 Jul 1;10(7):e0131979. doi: 10.1371/journal.pone.0131979 (PMC4488471; doi:10.1371/journal.pone.0131979)
Supplement: S2 Table — (PDF) [file pone.0131979.s007.pdf]

Table S2: Age-specific prevalences of dementia

	Bottino	Bottino adjusted	Lopes	Lopes adjusted	Herrera	Herrera adjusted	Scazufca	Ramos-Cerqueira	Magalhães
60-69	-	-	-	-	-	-	-	-	44.0
60–64	2.4	3.2	1.9	3.7		-	-	-	-
65–69	4.1	5.6	2.2	3.4	1.6	3.7	2.3	0.1	-
70-79	-	-	-	-	-	-	-	-	52.7
70–74	7.1	8.9	6.3	9.5	3.2	7.3	2.0	0.1	-
75–79	9.5	13.0	9.7	14.4	7.9	18.0	7.8	2.6	-
80-89	-	-	-	-	-	-	-	-	53.5
80–84	13.3	16.1	15.5	24.0	15.1	34.6	13.6	3.1	-
≥85	22.4*	40.7*	29.5*	37.6*	38.9	88.7	21.4	12.0	-
85-89	15.3	20.5	18.5	22.7	-	-	-	-	-
≥90	42.3	48.9	47.1	59.2	-	-	-	-	86.7

*Values calculated from the tables presented in the papers
